# Identification of a novel nonsense variant c.1332dup, p.(D445*) in the *LDLR* gene that causes familial hypercholesterolemia

**DOI:** 10.1038/hgv.2014.21

**Published:** 2014-11-20

**Authors:** Faisal A Al-Allaf, Mohammad Athar, Zainularifeen Abduljaleel, Abdellatif Bouazzaoui, Mohiuddin M Taher, Rakan Own, Ahmad F Al-Allaf, Iman AbuMansour, Zohor Azhar, Faisal A Ba-hammam, Hala Abalkhail, Abdullah Alashwal

**Affiliations:** 1 Department of Medical Genetics, Faculty of Medicine, Umm Al-Qura University, Makkah, Saudi Arabia; 2 Science and Technology Unit, Umm Al-Qura University, Makkah, Saudi Arabia; 3 Molecular Diagnostics Unit, Department of Laboratory and Blood Bank, King Abdullah Medical City, Makkah, Saudi Arabia; 4 College of Medicine, Alfaisal University, Riyadh, Saudi Arabia; 5 Department of Pediatrics, King Faisal Specialist Hospital and Research Centre, Riyadh, Saudi Arabia

## Abstract

Familial hypercholesterolemia (FH) is an autosomal dominant disease predominantly caused by a mutation in the low-density lipoprotein receptor (*LDLR*) gene. Here, we describe two severely affected FH patients who were resistant to statin therapy and were managed on an apheresis program. We identified a novel duplication variant c.1332dup, p.(D445*) at exon 9 and a known silent variant c.1413A>G, p.(=), rs5930, NM_001195798.1 at exon 10 of the *LDLR* gene in both patients.

## Introduction

Familial hypercholesterolemia (FH) is an autosomal dominant disorder that predisposes the patient to the development of coronary artery disease and sudden cardiac death. FH is generally caused by mutations in the low-density lipoprotein receptor (*LDLR*) gene, leading to reduced hepatic clearance of LDL from the blood. FH can also be caused by mutations in the apolipoprotein B (*APOB*) gene, which encodes the LDLR ligand. In addition, a pathogenic mutation in the proprotein convertase subtilisin/kexin type 9 (*PCSK9*) gene has been proposed to cause FH, indicating that the disease is genetically heterogeneous.^1–3^ Several studies have demonstrated that mutations in the *LDLR, APOB* or *PCSK9* genes can result in hypercholesterolemia.

The *LDLR* gene consists of 18 exons spanning 45 kb and encodes an 860-amino-acid precursor protein.^4^ In the majority of populations, the frequency of heterozygosity is less than 1:500, whereas the homozygous form is rare, occurring at a frequency of 1:1,000,000.^5^ Worldwide, there are more than 1,288 reported genetic variants in LDLR.^6–8^ The highest frequency of heterozygosity is observed in the South African population, with an incidence of less than 1:80.^9^ Other studies in the French-Canadian population found five common variants with a frequency of 1:270.^4,10^ This high frequency is attributed to the founder effect and consanguineous marriages.

Before this research, a genetic epidemiological study of the frequency of FH in the Saudi population had not been conducted. We expect the incidence of FH, secondary to the homozygous genotype in Saudi Arabia, to be relatively high, because of the increased rate of consanguineous marriages (over 54%). Consequently, many cardiovascular centers have been established across the country to provide adequate care and treatment for patients with heart diseases.^11^ The estimated number of affected heterozygote Saudi individuals ranges from 46,000 to 230,000 individuals. The majority of affected individuals may not be aware of the disease, as they remain asymptomatic until a severe myocardial infarction occurs, usually after the age of 40 years. The myocardial infarctions can be severe enough to cause sudden cardiac death or disabling cardiovascular morbidities. For the clinical diagnosis of FH, there are three sets of criteria used: the Simon Broome Register (UK), the Dutch Lipid Clinic Network (the Netherlands) and the MEDPED Program (USA). These criteria have been proven to identify FH in patients. The criteria are primarily based on age, blood cholesterol levels and evidence of clinical signs related to FH (xanthelasma, tendinous xanthomata and corneal arcus) and a family history of coronary heart disease (CHD). However, elevated blood cholesterol levels are commonly observed in individuals with nongenetic multifactorial hypercholesterolemia, which may lead to a misdiagnosis of FH in those individuals. Therefore, the use of molecular methods to characterize gene defects is necessary for an unequivocal FH diagnosis. Furthermore, when novel variants are identified, distinguishing between pathogenic variants and polymorphs is crucial for molecular confirmation. Further investigation into the pathogenicity of novel variants can be performed by standard functional analyses or by using new alternative bioinformatics methods to predict the putative effects of variants on protein function and stability.^12^

## Materials and methods

### Subjects

The analysis was performed in two patients diagnosed with homozygous FH. The patients are siblings whose family originates from a tribe that lives in the northern region of Saudi Arabia. Both patients are resistant to statin therapy and were on an apheresis program. Sample collection and studies were performed in accordance with the Research Ethics Committee’s regulation after the subjects were provided with informed consent. The enrollment criteria for the genetic screening of the patients were based on the Simon Broome register.^13^

### DNA analysis

Genomic DNA was isolated from EDTA-treated whole blood using the MagNA Pure Compact Nucleic Acid Isolation Kit I (Roche, Basel, Switzerland) according to the manufacturer’s instructions. Polymerase chain reaction (PCR) amplification of the *LDLR* gene (including the 18 coding exons and flanking intron regions), *APOB* gene (exon 26 of the *APOB* gene containing codons 3,475–3,592, which harbors three known pathogenic variant sites, R3500Q, R3500W and R3527Q) and the *PCSK9* gene (the 12 exons and flanking intron regions) were performed. Descriptions of the primers used for amplifying and sequencing the fragments are provided in Supplementary Table 1. PCR was performed with 100 ng genomic DNA using the HotStarTaq Plus DNA Polymerase Kit (Qiagen, Hilden, Germany) as follows: *Taq* polymerase was activated at 94 °C for 5 min, followed by 35 cycles of denaturing at 94 °C for 30 s, annealing at 61–64 °C (Supplementary Table 1) for 30 s, extension at 72 °C for 45 s and final extension at 72 °C for 5 min. The amplified products were separated on an agarose gel to ensure the size and quality of the band. The PCR products were purified using magnetic beads with the Agencourt AMPure XP kit (Beckman Coulter, Brea, CA, USA). The purified products were used as templates for direct sequencing with a BigDye Terminator v3.1 cycle sequencing ready reaction kit (Applied Biosystems, Foster City, CA, USA). The sequencing reaction products were purified using the BigDye X-terminator purification kit (Applied Biosystems) followed by capillary electrophoresis in an ABI 3500 Genetic analyzer (Applied Biosystems). The final analysis was performed using the Sequence Analysis Software v5.4 (Applied Biosystems).

## Results

**Figure 1:**
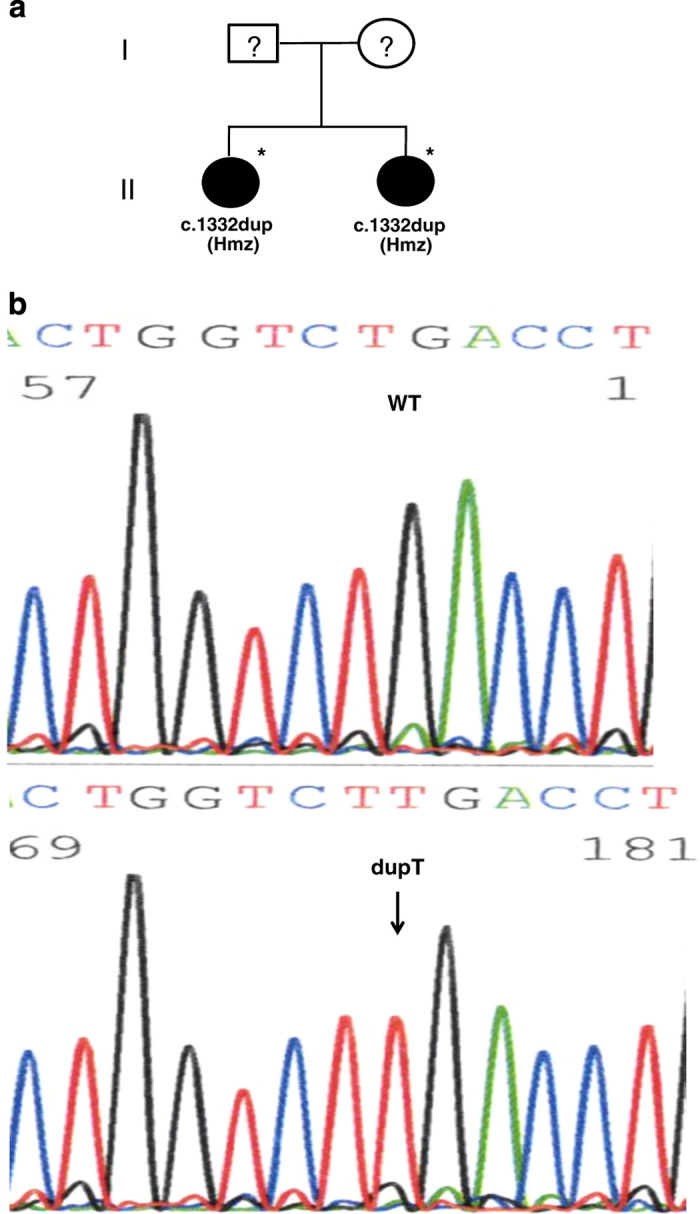
Novel nonsense variant p.(D445*) identified at exon 9 of the *LDLR* gene. (**a**) Pedigree of the patient family. (**b**) Representative DNA sequence from the control individuals (wild type; WT) and patients. ?=NOT available for DNA analysis. *=Index case.

The genomic analysis revealed a novel duplication variant c.1332dup, p.(D445*) at exon 9 of the *LDLR* gene in the two patients (Figure 1). Both patients were determined to be homozygous for this variant, which expresses a premature stop codon at position 445 in exon 9 of the *LDLR* gene. Consequently, a truncated protein is produced, which is a defective LDL receptor. In addition to this novel nonsense variant, we also identified a known silent variant c.1413A>G, p.(=), rs5930, NM_001195798.1 in both patients at exon 10 of the *LDLR* gene (Table 1). Figure 1a shows the pedigree of the family (the parents and the first-degree relatives were not available for DNA analysis at the time of the study). Table 1 also shows the total and LDL cholesterol levels with values reaching 18.11 and 15.05 mmol/l in patient one and 15.13 and 12.98 mmol/l in patient two. These values are considered very high compared with the optimal level, which is <2.59 mmol/l (LDL cholesterol). This confirms the loss of LDLR function because of the nonsense variant present in the DNA sequence.

## Discussion

To date, there are more than 1,288 LDLR variants reported in FH patients worldwide that are regarded as pathogenic variants.^8,14–16^ We identified a novel duplication variant (c.1332dup) at exon 9 of the *LDLR* gene. This duplication generates a defective LDL receptor because of the premature stop codon at position 445 in exon 9 of the human *LDLR* gene. No other variants were observed in the *LDLR*, *APOB* and *PCSK9* genes.

This novel nonsense variant is localized in the EGF precursor homology domain. Theoretically, the membrane-spanning domain and the remainder of the LDLR protein cannot be synthesized; therefore, the truncated protein may be degraded by the proteasome machinery. This hypothesis agrees with the patient phenotypes, that is, both patients were resistant to statin therapy and were on an apheresis program. Furthermore, an earlier study determined that up to 54% of LDLR variants that resulted in FH were localized in the epidermal growth factor precursor (EGFP) homology domain.^16^ Marduel *et al.*^7^ reported nine nonsense variants that were classified as FH-causing variants because of the synthesis of a truncated protein. In addition, the lipid profile of our patients supports the loss of LDLR function, as the analysis of the total and LDL cholesterol levels revealed an extremely high concentration of more than 12 mmol/l. Interestingly, the levels of LDL and total cholesterol in both our patients are higher compared with the maximal level of the FH group that was presented in Marduel *et al.*^7^ This suggests that it is necessary to establish a validated LDL concentration cut-off for the clinical diagnosis of FH in Saudi Arabia. In conclusion, the segregation pattern of the variant is consistent with the lipid profile (Figure 1 and Table 1), suggesting a more severe FH phenotype when the variant is in the homozygous state.

## Figures and Tables

**Table 1:** Characteristics of the studied patients with the novel nonsense variant p.(D445*) at exon 9 of the *LDLR* gene

*Mutations and parameters*	*Patient 1*	*Patient 2*	*Normal range (mmol/l)*
Nonsense variant in exon 9 of the *LDLR* gene	c.1332dup, p.(D445*)	c.1332dup, p.(D445*)	—
Silent variant in exon 10 of the *LDLR* gene	c.1413A>G, p.(=) rs5930, NM_001195798.1	c.1413A>G, p.(=) rs5930, NM_001195798.1	—
Sex (M/F)	F	F	—
Age (year)	20	22	—
Total cholesterol (mmol/l)	18.111	15.133	3.8–7.5
LDL-C (mmol/l)	15.05	12.98	2.6–5.2
Triglyceride (mmol/l)	1.01	1.43	0.3–1.6
HDL-C (mmol/l)	0.95	1.12	0.8–2.1
Medication	Ezetimibe, Acetazololamide, Simvastain	Ezetimibe, Simvastain	—
CHD history in parents	Yes	Yes	—

Abbreviations: CHD, coronary heart disease; F, female; HDL-C, high-density lipoprotein-cholesterol; LDL-C, low-density lipoprotein-cholesterol; LDLR, low-density lipoprotein receptor; M, male.
