# Supplementary material for: Identification of a novel nonsense variant c.1332dup, p.(D445*) in the LDLR gene that causes familial hypercholesterolemia
Source: Hum Genome Var. 2014 Nov 20;1:14021–. doi: 10.1038/hgv.2014.21 (PMC4785512; doi:10.1038/hgv.2014.21)
Supplement: Supplementary Table 1 [file hgv201421-s1.doc]

**Supplementary Information**

**Supplementary Table 1.** Primers and conditions used for PCR amplification and sequencing of the DNA fragments.
